# Important Methodological and Statistical Concerns Regarding the Association of Factor V Leiden and MTHFR Polymorphisms With COVID‐19 Outcomes

**DOI:** 10.1002/iid3.70524

**Published:** 2026-09-09

**Authors:** Aditya K. Panda

**Affiliations:** ^1^ ImmGen EvSys Lab, BT‐113, Department of Biotechnology Berhampur University Berhampur Odisha India; ^2^ Centre of Excellence on Bioprospecting of Ethno‐pharmaceuticals of Southern Odisha (CoE‐BESO) Berhampur University Berhampur Odisha India

Dear Editor,

We read with great interest the recently published article by Çıralıoğlu et al., entitled “A Case‐Control Study of Factor V Leiden G1691A and MTHFR A1298C Polymorphisms and Clinical Outcomes in Patients With COVID‐19” [1]. In this study, the authors investigated an interesting question concerning the possible role of genetic variants linked to coagulation abnormalities and clinical outcomes in COVID‐19. Most interestingly, the authors examined both individual polymorphisms and their combined occurrence in the clinical phenotype of COVID‐19. However, I observed several methodological and statistical issues that require clarification, as they may substantially influence the interpretation of the reported associations.

First, the authors have not tested the distribution of genotypes in accordance with or not Hardy Weinberg Equilibrium (HWE) in healthy subjects. The distribution of MTHFR A1298C genotypes was deviated from HWE. The control group consisted of 184 AA and 116 AC individuals, with no CC individuals. A conventional HWE calculation gives a highly significant deviation (*χ* ^2 ^= 17.23, *p* = 0.00003). In contrast, the FVL G1691A genotypes in healthy controls were in accordance with HWE (*χ* ^2 ^= 1.44, *p* = 0.22). This issue is particularly important in a genetic case‐control study because substantial deviation of the genotype distribution from HWE in healthy controls may be attributed to several factors, including genotyping error, population stratification, or selection effects [2, 3]. The manuscript neither includes any HWE analysis nor discusses this deviation. We strongly believe that the authors must disclose HWE assessment and clarify whether genotyping quality‐control procedures, duplicate samples, negative controls, and independent genotype confirmation were performed in the reported investigation.

Second, the genetic analysis was poorly reported in this case‐control association study. Table 1 presents the genotype frequencies and *χ* ^2^ *p* values, as well as the effect size (phi values). However, important parameters in a genetic association study, such as allele frequencies, odds ratios (ORs), and 95% confidence intervals, are lacking. Using the genotype data from Table 1, the OR for FVL GA versus GG is 1.96 (95% CI 1.16–3.25), whereas the corresponding OR for MTHFR AC versus AA is 0.48 (95% CI 0.31–0.74). Thus, the presentation of statistical analysis of genetic case control study in terms of OR and 95% CI gives both direction and magnitude of the associations, which are considerably more informative than *p* values alone [4]. The absence of homozygous variant genotypes hinders the evaluation of additive, recessive, and homozygote‐specific effects. Reporting allele frequencies and explicitly defining the genetic model would substantially improve interpretability.

Although this study presents potential findings regarding the relationship between FVL, MTHFR, and COVID‐19‐related outcomes, the reliability of these findings is substantially affected by the issues noted above. I believe that clarification and, where possible, reanalysis of the genetic data would strengthen the scientific value of this work and help readers appropriately interpret the potential contribution of FVL and MTHFR polymorphisms to COVID‐19‐related outcomes.

## Author Contributions

**Aditya K. Panda:** conceptualization, supervision, writing – review and editing, writing – original draft.

## Funding

The author has nothing to report.

## Conflicts of Interest

The author declares no conflicts of interest.

## Data Availability

Data sharing not applicable to this article as no datasets were generated or analyzed during the current study.
